# Environmentally-induced sperm RNAs shape placentation and fetal growth

**DOI:** 10.21203/rs.3.rs-9306272/v1

**Published:** 2026-04-17

**Authors:** Duane Gischewski Pereira, Raquel Santana da Cruz, Meghali Joshi, Lu Jin, Elaine Chen, Hannah De Los Santos, Lili Rodgers, Caroline Crone, Rong Hu, Andrew D. Holmes, Upasna Sharma, Susana Galli, Sonia de Assis

**Affiliations:** 1 Department of Oncology, Lombardi Comprehensive Cancer Center, Georgetown University, Washington, DC, USA; 2 Department of Molecular, Cell and Developmental Biology, University of California Santa Cruz, CA, USA.; 3 Department of Biochemistry and Molecular & Cell Biology, Georgetown University, Washington, DC, USA

## Abstract

Sperm RNAs are environment-sensitive and mediate paternally-induced epigenetic inheritance of traits. The sperm RNA cargo is delivered to the oocyte during fertilization but precise mechanisms by which the ‘epigenetic memory’ it carries alters embryonic development to shape the progeny phenotypes remains poorly understood. Here, we used two mouse paradigms of male pre-conception experiences to study whether environmentally-induced sperm RNAs regulate placenta development. Using zygotic injections, we show that the sperm RNA load from DDT-treated and obese males impacts placenta function and fetal growth in opposite ways. Mechanistically, these environmentally-driven sperm RNAs disrupt early embryonic development and cell lineage specification. They also dictate the mature placenta cellular composition, nutrient stores, vascularization and imprinted genes landscape. Our findings provide direct evidence that sperm RNAs shape placentation, offering a mechanism by which fathers influence their progeny beyond their shared genome. These data also highlight the role of paternal non-genetic factors in pregnancy outcomes.

## Introduction

Mendelian genetics account for the majority of inherited traits, including disease predisposition, but not all. From lower forms of life to humans^1–4^, there is evidence that intergenerational epigenetic inheritance occurs and explains a portion of phenotypes transmitted via the germline.

Sperm small non-coding (snc)RNAs have been shown to mediate epigenetic inheritance of traits through the male gamete in different animal models.^2,5–7^ The mammalian sperm acquires its mature sncRNAs cargo following testicular exit and its transit in the epididymis. These sncRNAs can be of somatic origin, acquired via epididymosomes or sperm born via cytoplasmic droplets or mitochondrial-derived RNA transcripts.^8–10^ Importantly, the sperm RNA load is sensitive to environmental insults.^7,11–13^ Although is recognized that the sperm RNA cargo is delivered to the oocyte during fertilization^14,15^, the precise mechanisms by which this ‘epigenetic memory’ in sperm RNAs alters the course of embryonic and fetal development to shape the progeny’s phenotypes remains poorly understood.

In eutherian mammals, the placenta connects the fetus to the mother to ensure nutrient and gas exchanges and hormone production necessary for maintenance of pregnancy and optimal fetal development.^16,17^ This temporary organ also serves as a physical and immunological barrier that protects the fetus from harmful agents and pathogens.^18^ The process of placentation begins in early development and is highly orchestrated: At the blastocyst stage, embryonic cells undergo their first fate decision and differentiate into either the inner cell mass (ICM) or the trophectoderm (TE) cell lineages. TE cells give rise to the placenta while ICM cells further differentiate into the epiblast and the primitive endoderm, which will form the embryo proper and the yolk sac, respectively.^16,19^ The TE allows the embryo to implant into the maternal endometrium^17^. In both mice and humans, implantation triggers stromal cells in the maternal uterine lining to undergo a process of decidualization.

After implantation of the mouse blastocyst, TE cells start to differentiate, generating multiple specialized placenta trophoblast subpopulations.^20^ TE cells also form the extra-embryonic ectoderm and the ectoplacental cone. At gastrulation, the epiblast gives rise to the extra-embryonic mesoderm lineage and forms the allantois, the amnion and chorion. The chorio-allantoic fusion allows extra-embryonic mesoderm-derived blood vessels to invaginate into the chorionic trophoblast layer. These fetal blood vessels and trophoblast-lined maternal blood sinuses form the main structure of the placental labyrinth that enable nutrient and gas exchange between the maternal and fetal blood starting at mid-gestation.^16^ In addition to the labyrinth zone, the fully mature mouse placenta consists of two other major areas: the decidua, the maternal layer that connects to the uterus, and a middle layer called junctional zone that contains cells such as spongiotrophoblasts and glycogen trophoblasts (GlyT), which provide hormonal and nutritional support to the fetus.^17^

Defective placentation impairs fetal growth and can have long-lasting effects on the progeny health.^21,22^ Not surprisingly, a role for the placenta in epigenetic inheritance has been proposed.^21^ Because of the close relationship between mother and child in pregnancy, studies link maternal exposures in pregnancy to placenta development and function.^23,24^ Despite experimental evidence indicating that placentation is heavily driven by paternal factors, particularly imprinted genes^22,25–27^, our understanding of the impact of paternal pre-conception experiences on placentation is limited, though evidence is emerging.^28–34^

Here, we demonstrate that paternal experiences and environmentally-induced sperm non-coding RNAs shape placenta development. We show that sperm RNA cargo from male mice treated with the legacy pesticide dichlorodiphenyltrichloroethane (DDT)—which is linked to low birth weight in humans^35,36,^ and mice^32^—is functionally linked to placenta inefficiency and fetal growth restriction. In contrast, obesity-induced sperm RNAs increased placenta efficiency and fetal growth. Mechanistically, the sperm RNA load from DDT-exposed or obese males disrupts early embryonic development, alters the expression of imprinted genes and cellular composition in the mature placenta. Collectively, our findings provide compelling evidence that sperm RNAs shape placentation and offer a mechanism by which fathers impact progeny health beyond their shared genome.

## Results

### DDT-induced sperm RNAs impair placenta function and fetal growth

Epidemiological reports link paternal pre-conception exposures to environmental endocrine disruptors^35,36,37,38^ to offspring’s low birth weight. These findings are supported by rodent models showing that the progeny of DDT-exposed males are born smaller and exhibit metabolic dysfunction^32,39^ in adulthood, phenotypes linked to placental abnormalities.^32^

**Extended data Fig.1.**
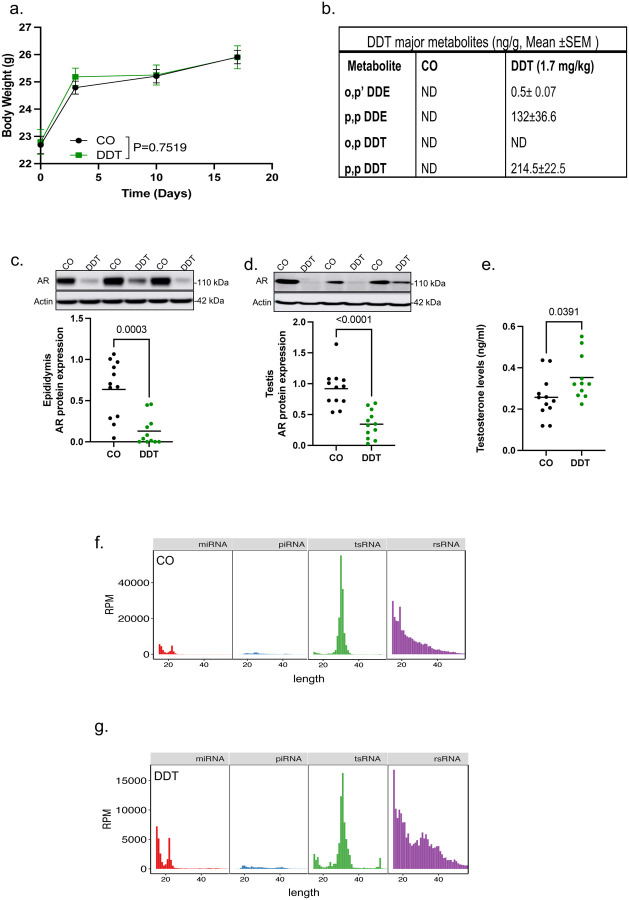
Males were exposed to a solution of DDT (1.7mg/kg) or vehicle-control (CO) for 17 days. (a) Longitudinal bodyweight of males during treatment with a DDT or CO solution (n=30 males/group). (b) Hepatic levels of DDT main metabolites in CO or DDT-treated mice (n=2 of 5 pooled liver samples/group) by GC-MS/MS 24h after the last treatment. ND, non-detectable. (c-d) Androgen receptor (AR) protein levels (representative image and densitometry) in (c)epididymis or (d) testis of CO or DDT treated male mice (n=10–12/group). (d) Levels of blood testosterone in vehicle-control (CO) or DDT treated male mice measured by ELISA (n=11–12/group). (f-g) Read count (RPM) summary and length distributions of different sncRNA subtypes in sperm RNA-seq analysis in (f) CO or (g) DDT-treated male mice (n=4/group). Data shown as mean–SEM (a) or mean (horizontal bars in scatter plots in c,d,e). Data were analyzed by two-way ANOVA (a) or t-test (c,d,e).

**Fig 1.**
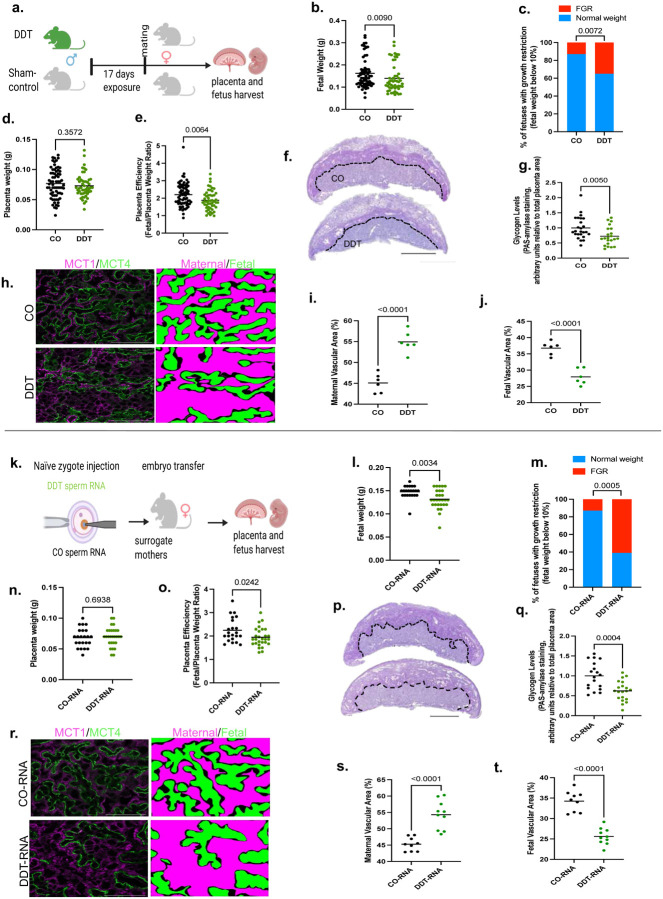
DDT-induced sperm RNAs impair placenta function and fetal growth. (a) Experimental design: Males were exposed to a solution of DDT (1.7mg/kg) or vehicle-control (CO) for 17 days and then mated to unexposed females. Pregnant dams were euthanized on E13.5 for placenta and fetal collection. (b) Fetal weight (grams, n=51–69, 7–9 litters). (c) Percentage of fetal growth restriction (FGR, 10th percentile fetal weight cut-off of the control group). (d) Placenta weight (grams, n=51–69, 7–9 litters). (e) Placenta efficiency (gram of fetus per gram of placenta, n=51–69, 7–9 litters). (f) Representative pictures of PAS (dark magenta above dotted line, glycogen marker, bar=1mm) stained placentas. (g) Placenta glycogen level quantification (PAS-amylase staining, normalized by placenta area, n=22–25, 6–9 litters). (h) Representative picture of placental labyrinth zone stained for MCT1 (magenta, syncytiotrophoblast-I) and MCT4 (green, syncytiotrophoblast-II), demarcating the maternal blood sinusoids and fetal vessels, respectively (bar=50um). (i-j) Quantification of maternal (i) and fetal maternal (j) blood spaces (n=6, 3 litters). (k) Experimental design: Naïve mouse embryos at the zygote stage were injected with the entire RNA load from the sperm of either CO or DDT-exposed males. Injected zygotes were transferred into recipient female mice to produce offspring. Pregnant dams were euthanized on E13.5 for placenta and fetal collection. (l) Fetal weight (grams, n=23–28, 6–7 litters) (m) Percentage of fetal growth restriction (10th percentile fetal weight cut-off of the control group) (n) Placenta weight (grams, n=23–28, 6–7 litters). (o) Placenta efficiency (gram of fetus per gram of placenta, n=23–28, 6–7 litters). (p) Representative pictures of PAS (dark magenta above dotted line, glycogen marker, bar=1mm) stained mouse placentas. (q) Placenta glycogen level quantification (PAS-amylase staining, normalized by total placenta area, n=18–20, 5–6 litters). (r) Representative picture of placental labyrinth zone stained for MCT1 (magenta, syncytiotrophoblast-I) and MCT4 (green, syncytiotrophoblast-II), demarcating the maternal blood sinusoids and fetal vessels, respectively (bar=50um). (s-t) Quantification of maternal (s) and fetal maternal (t) blood spaces (n=9–10, 6 litters). Data shown as mean (horizontal bars in scatter plots in b,d,e,g,i,j,l,n,o,q,s,t) or percentage (c,m). Data were analyzed by t-test or Mann-Whitney test (b,d,e,g,i,j,l,n,o,q,s,t) or Fisher’s exact test (c,m).

To further understand the impact of paternal environmental experiences on placentation and fetal growth, we treated adult male mice with DDT solution or the vehicle-control (CO) for 17 days (Fig.1a, Extended data Fig.1a–b). Like other androgen antagonists^40^, DDT competes with circulating testosterone for androgen receptor (AR) binding, resulting in a negative feedback loop in the brain. This causes an increase in baseline testosterone levels and AR suppression^40,41^. Consistent with that, we found that epididymal and testicular AR expression were reduced while circulating testosterone levels were increased in DDT-exposed males compared to the CO group (Extended data Fig. 1c–e).

Next, we mated DDT-exposed or CO males with healthy untreated female mice overnight. Pregnant dams were euthanized and both placenta and fetal tissues harvested on embryonic day (E)13.5, after placentation is complete in mice^17^ (Fig.1a). We observed fetal growth restriction in progeny of DDT exposed males, with increased proportion of fetuses below the 10% for weight compared to controls (Fig.1 b–c). While there were no differences in placental weight (Fig.1d), we found a significant decrease in placenta efficiency (Fig.1e) —defined as grams of fetus produced per gram of placenta—in pregnancies from mating with DDT-treated males.

**Extended data Fig.2.**
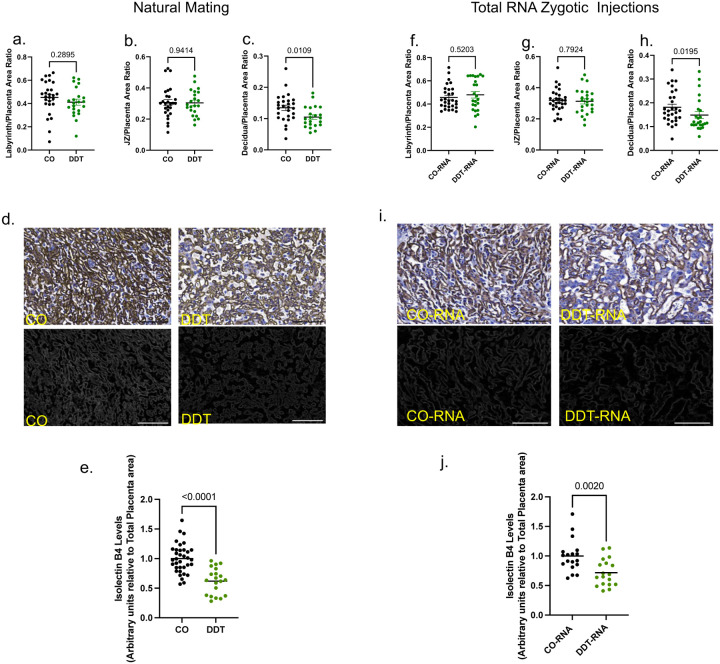
(a-c) Males were exposed to a solution of DDT (1.7mg/kg) or vehicle-control (CO) for 17 days and then mated to unexposed females. Pregnant dams were euthanized on E13.5 for placenta and fetal collection. Layer-specific placenta surface area normalized to total placenta area and shown by study and group: (a) Labyrinth zone, (b) Junctional zone (JZ) and (c) Decidua. Placenta images were analyzed via image J software. Data shown as mean–SEM (horizontal bars in scatter plots), n = 22–27/group 6–7 litters). (d) Representative pictures of placenta labyrinth zone showing isolectin B4 (IB4) staining (brown, fetal vessel marker, bar=100um). (e) Placenta fetal capillary quantification (IB4, normalized by placenta area, n=20–35, 6–9 litters) Data shown as mean (horizontal bars in scatter plots). (f-h) Naïve mouse zygotes were injected with the entire RNA load from the sperm of either CO or DDT-exposed males. Injected zygotes were transferred into recipient female mice to produce offspring. Pregnant dams were euthanized on E13.5 for placenta and fetal collection. Layer-specific placenta surface area normalized to total placenta area and shown by study and group: (f) Labyrinth zone, (g) Junctional zone (JZ) and (h) Decidua. Placenta images were analyzed via image J software. Data shown as mean–SEM (horizontal bars in scatter plots, n = 24–29 /group 7–8 litters). (i) Representative pictures of placenta labyrinth zone showing IB4 staining (brown, fetal vessel marker, bar=100um). (j) Placenta fetal capillary quantification (IB4, normalized by total placenta area, n=18, 5–6 litters; Data shown as mean (horizontal bars in scatter plots). All data were analyzed by t-test.

Morphological analysis revealed a significant decrease decidua area of the DDT group placentas, but no changes in labyrinth or junctional zones, compared to controls (Extended data Fig. 2a–c). The GlyT within the junctional zone, particularly invasive GlyT migrating to the decidua, provides energetic and hormonal support to the fetus^17^. Quantification of placental glycogen content, revealed a significant decrease in placental glycogen levels in the DDT group compared to controls (Fig.1f–g). In parallel, we evaluated the structure of the fetal-placental vasculature in the labyrinth zone, where the exchange of nutrients and waste between the maternal and fetal circulations occurs. The labyrinth architecture was disrupted in placentas of DDT offspring with reduced fetal vasculature and dilated maternal spaces (Fig.1h–j, Extended data Fig. 2d–e).

The sperm RNA load, which is abundant in small non-coding RNAs, is modulated by the paternal environmental experiences and can transmit phenotypes to the progeny.^5,7,11,13^ To assess whether sperm RNAs of DDT-treated males are functionally linked to impaired placenta and fetal development, we performed zygote injection using the total RNA load extracted from mature sperm of either DDT or CO males. Injected embryos were then transferred into surrogate dams for timed placenta and fetal tissue collection at E13.5 (Fig.1k). Remarkably, zygotic injection of the sperm RNA load from DDT-exposed males (DDT-RNA), recapitulated the original phenotype observed in DDT offspring. Fetuses in the DDT-RNA group weigh less than those in the CO- RNA group (Fig. 1l), with a significant increase in fetal growth restriction (Fig.1m). Compared to controls, placenta efficiency, but not weight, was significantly reduced in DDT-RNA group (Fig.1n–o). Further replicating the original DDT group phenotypes, decidua area (Extended Fig.2f–h) and glycogen levels (Fig.1 p–q) were significantly decreased in placenta of DDT-RNA offspring. DDT sperm RNA zygotic injection also replicated the abnormal labyrinth architecture with DDT-RNA placentas showing reduced fetal vasculature and expanded maternal sinuses compared to CO-RNA placentas (Fig.1r–t, Extended Fig.2i–j).

These results indicate that disruption of the decidualization process, placenta reduction in glycogen producing cells and vascular branching insufficiency impair nutrient and gas transfer to support fetal energy needs, resulting in fetal growth restriction in both DDT and DDT-RNA offspring.

### Pre-conception paternal exposure to DDT modulates the sperm non-coding RNA content

**Fig 2.**
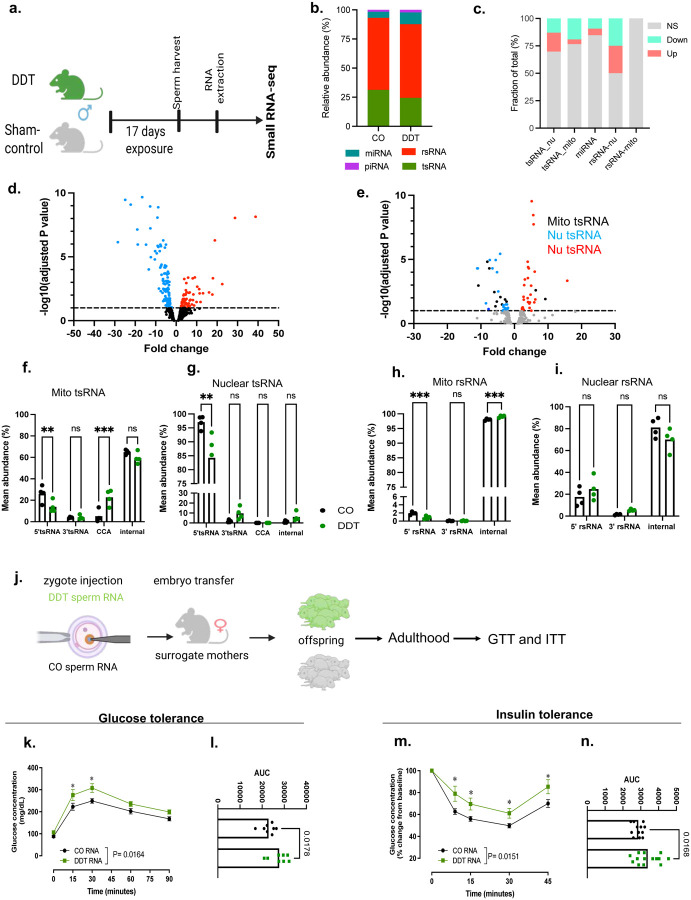
Pre-conception paternal DDT exposure modulates the sperm non-coding RNA content and transfer susceptibility to disease to the progeny. (a) Experimental Design: Males were exposed to a solution of DDT (1.7mg/kg) or vehicle-control (CO) for 17 days. Sperm was collected at the end of the exposure period and used for RNA extraction and for small RNA-seq analysis. (b) Relative distribution of sncRNA subtypes in cauda spermatozoa from sham control (CO) and DDT-exposed mice assessed via small RNA-seq (miRNA: microRNA: tsRNA: tRNA-derived small RNAs: rsRNA; rRNA-derived small RNAs; piRNA: piwi RNA). (c) Subtype-specific fraction (percentage of the total reads) showing statistically significant up- or down-regulated cauda sperm sncRNAs. (d-e) Volcano plot representation of differentially expressed miRNAs, mitochondrial (mito) and nuclear tsRNAs. (f-i) Fragmentation patterns of (f-g) mito and nuclear tsRNAs or (h-i) mito and nuclear rsRNAs in cauda sperm from CO and DDT-exposed mice (n=4). (j) Experimental design: Naïve mouse embryos at the zygote stage were injected with the entire RNA load from the sperm of either CO or DDT-exposed males. Injected zygotes were transferred into recipient female mice to produce offspring. (k-n) Metabolic function in offspring was assessed at 20–24 weeks of age: (k-l) Glucose tolerance test (GTT) and (m-n) Insulin Tolerance Test (ITT) in CO or DDT-RNA offspring (n=8). Data shown as percentage (b,c) or as mean (f-i, k-n). Fragmentation patterns data (f-i), GTT (k) and ITT (m) were analyzed by two-way ANOVA, followed by Holm-Sidak’s test. AUC data (l,n) were analyzed by t-test or Mann Whitney’s test. *P < 0.05, **P < 0.01, ***P < 0.001.

Because our data indicate that sperm RNAs mediate observed DDT-induced phenotypes in placentation and fetal growth, we next examined the impact of DDT exposure on the mature sperm RNA content by performing small RNA-seq analysis (Fig. 2a). The distribution of the major small RNA species is in line with previous reports,^11,13^ with rRNA and tRNA-derived RNAs (rsRNA and tsRNA) being the most abundant small RNAs, followed by miRNAs and piRNAs in both groups (Fig. 2b). We found a subset of differentially expressed miRNA (Fig. 2c–d), but not piRNAs. Differential expression analysis also showed significantly upregulated or down-regulated mitochondrial and nuclear rsRNA and tsRNA in sperm of DDT-exposed males compared to the CO (Fig. 2c,e). Further analysis of tRNA fragmentation patterns revealed a significant decrease in the abundance of 5’-end nuclear and mitochondrial-derived tsRNAs in sperm of DDT-exposed males. We detected higher abundance in CCA-end mitochondrial-derived tsRNA fragments in sperm of DDT-exposed males compared to CO (Fig. 2f–g). The patterns of fragmentation for mitochondrial-derived, but not nucleus-derived, rsRNAs were also altered in sperm of DDT-exposed males relative to CO, with a significant decrease in the proportion of 5’-end and an increase in the proportion of internal rsRNA fragments (Fig. 2h–i).

### The Sperm RNA cargo from DDT exposed males transfer susceptibility to disease to the progeny

**Extended data Fig.3.**
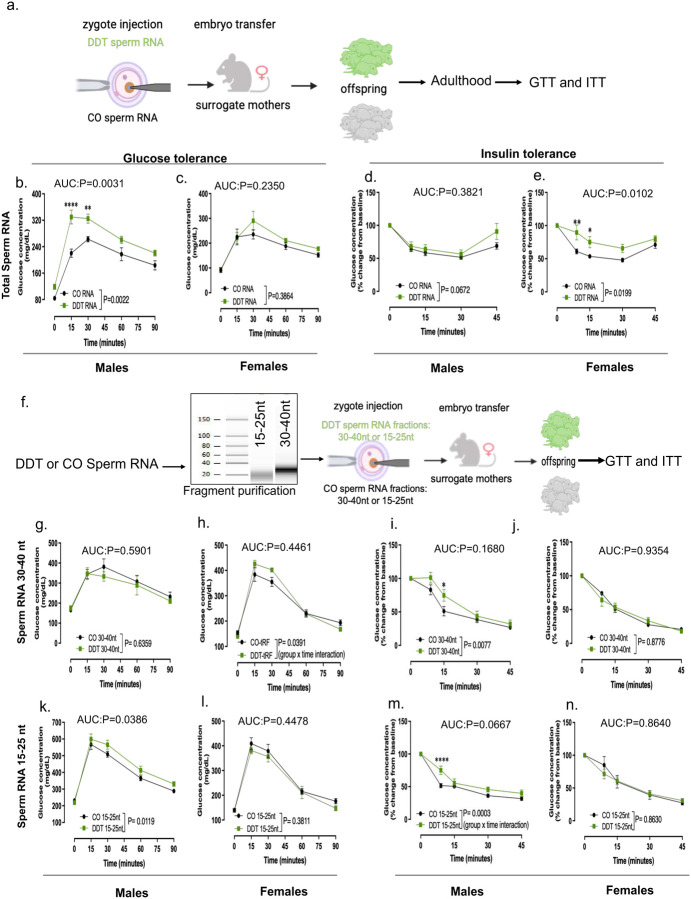
Naïve mouse embryos at the zygote stage were injected with the entire RNA load (a-e) or specific RNA fractions (f-n) from the sperm of either CO or DDT-exposed males. Sperm fractions containing the 30–40 nt or 15–25nt RNA fragments were purified using gel electrophoresis and gel excision with representative integrity and size analysis shown in f. Injected zygotes were transferred into recipient female mice to produce offspring. Metabolic function in offspring was assessed at 20–24 weeks of age: (b,c,g,h,k,l) Glucose tolerance test (GTT) and (d,e,i,j,m,n) Insulin Tolerance Test (ITT) in male (b,g,k, and d,i,m) and female (c,h,l and e,j,n) offspring. Data shown as mean–SEM, n=4–8 animals/group. Data were analyzed by two-way ANOVA, followed by Holm-Sidak’s test (b-e, g-n). AUC data was analyzed by t-test or Mann Whitney’s test.

Human cohort studies link parental exposures to DDT and environmental toxins to chronic diseases such obesity and cancer in the progeny.^42–44^ In agreement with these findings, rodent models show that paternal DDT exposure^32,39^ increases metabolic dysfunction in the offspring. To examine whether the sperm RNA cargo of DDT-treated males could transmit these phenotypes to the progeny, the RNA load extracted from mature sperm of either DDT-exposed or CO male mice was injected into naïve mouse embryos and transferred into surrogate dams to produce the DDT-RNA or CO-RNA offspring (Figure 2j, Extended data Fig. 3a). Consistent with results on placentation and fetal growth, DDT-RNA progeny showed impaired metabolic function compared to the CO-RNA group (Figure 2 k–n, Extended data Fig. 3b–e).

**Extended data Fig.4.**
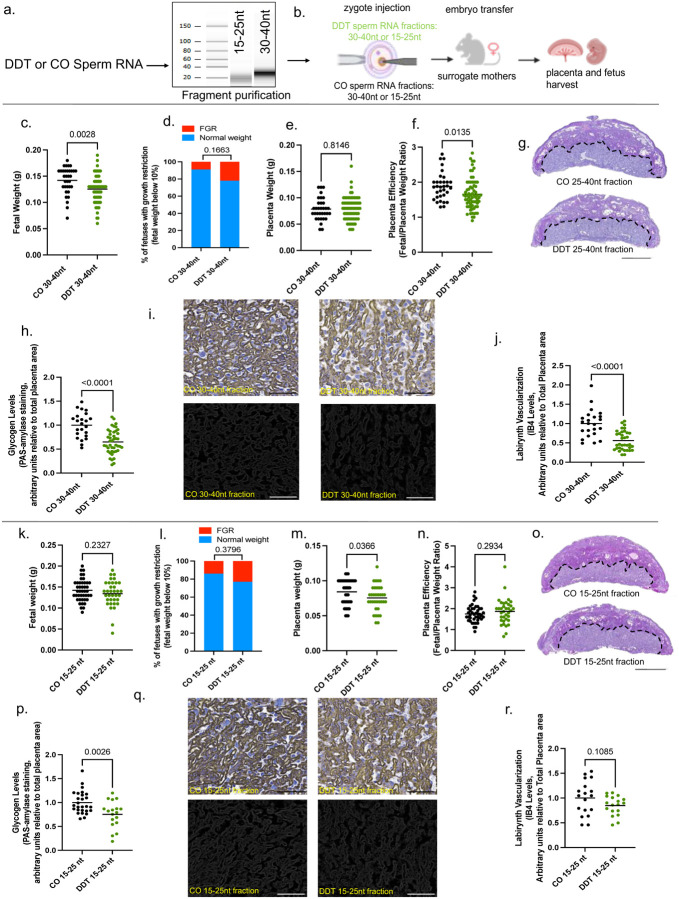
(a) Sperm fractions containing the 30–40nt or 15–25nt RNA fragments were purified using gel electrophoresis and gel excision with representative integrity and size analysis is shown. (b) Naïve mouse embryos at the zygote stage were injected with either the 30–40 nt or 15–25nt RNA fraction from sperm of CO or DDT-exposed males. Injected zygotes were transferred into recipient female mice to produce offspring. Pregnant dams were euthanized on E13.5 for placenta and fetal collection. (c-j) 30–40nt RNA fraction zygote injection: (c) Fetal weight (grams, n=33–69, 12–13 litters), (d) percentage of fetal growth restriction (10th percentile fetal weight cut-off of the control group), (e) placenta weight (grams, n=33–69, 12–13 litters), (f) Placenta efficiency (gram of fetus per gram of placenta, n=33–69, 12–13 litters). (g)Representative pictures of PAS-amylase staining (dark magenta above dotted line, glycogen marker, bar=1mm) in mouse placenta. (h) Placenta glycogen level quantification (PAS-amylase staining, normalized by placenta area, n=23–43 9–12 litters). (i) Representative pictures of placenta labyrinth zone showing IB4 staining (brown, fetal vessel marker, bar=100 um). (j) Placenta fetal capillary quantification (IB4, normalized by placenta area, n=23–35 8–11 litters). (k-r) 15–25nt RNA fraction zygote injection: (k) Fetal weight (grams, n=35–43,10 litters), (l) percentage of fetal growth restriction (10th percentile fetal weight cut-off of the control group), (m) placenta weight (n=35–43,10 litters), (n) Placenta efficiency (n=35–43,10 litters). (o) Representative pictures of PAS-amylase staining (dark magenta above dotted line, glycogen marker, bar=1mm) in mouse placenta. (p) Placenta glycogen level quantification (PAS-amylase staining, normalized by placenta area, n=18–27, 8–10 litters). (q) Representative pictures of placenta labyrinth zone showing IB4 staining (brown, fetal vessel marker, bar=100 um). (r) Placenta fetal capillary (IB4, normalized by placenta area, n=18–18, 8–10 litters). Data shown as mean (horizontal bars in scatter plots) or percentage. All data was analyzed by t-test or Mann Whitney’s test (c,e,f, h, j, k, m,n,p,r) or Fisher’s exact test (d,l).

Different classes of sperm small RNAs^1,5,13^ have been implicated in the transfer of information from fathers to offspring.^5,7,13^ and our data show differential expression of miRNA, tsRNA and rsRNAs in sperm of DDT-treated males (Fig. 2c–e). To establish which sperm small RNA subtypes may be functionally responsible for placental and progeny’s phenotypes, we purified the 15–25nt and 30–40nt sperm RNA fractions. The 15–25nt fraction contains the majority of miRNAs but also harbors piRNAs and smaller tsRNAs and rsRNAs. On the other hand, the 30–40nt sperm RNA fraction is composed of mostly tsRNAs and rsRNAs, but may also contain larger piRNAs (Extended data Fig. 1f–g). We next performed zygote injection using the sperm RNA fractions from either DDT-exposed or CO male mice. Injected embryos were transferred into surrogate dams to produce offspring or for timed placenta and fetal tissue collection at E13.5 (Extended data Fig.3f, Extended data Fig. 4a–b).

Although offspring generated with either the DDT-30–40nt or 15–25nt sperm fractions (Extended data Fig. 3f) showed slightly worse metabolic homeostasis compared to controls (with sex-specific differences depending on the fraction), neither fraction completely replicated the metabolic effects observed in DDT or DDT-RNA offspring (Extended data Fig. 3g–n).

**Extended data Fig.5.**
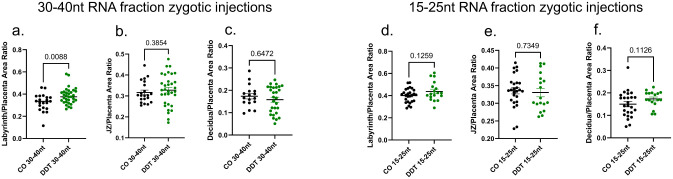
(a-f) Naïve mouse zygotes were injected with RNA fractions (30–40nt or 15–25nt) from the sperm of either CO or DDT-exposed males. Injected zygotes were transferred into recipient female mice to produce offspring. Pregnant dams were euthanized on E13.5 for placenta and fetal collection. Layer-specific placenta surface area normalized to total placenta area and shown by study and group: (a,d) Labyrinth zone, (b,e) Junctional zone (JZ) and (c, f) Decidua. Placenta images were analyzed via image J software. Data shown as mean±SEM (horizontal bars in scatter plots), n = 17–33, 9–12 litters (a-c), n = 17–26, 8–10 litters/group (d-f). Data were analyzed by t-test.

In alignment with the findings on the progeny phenotypes, neither sperm RNA fraction from DDT-exposed males fully recapitulated the placenta traits on their own (Extended data Fig.4) in contrast with zygotic injection of the total sperm RNA load from the same males. Although fetuses in the DDT-30–40nt sperm RNA fraction group weighed significantly less compared to the CO group, the proportion of fetuses below the 10% for weight did not differ between groups (Extended data Fig.4c–d). Placenta efficiency, but not weight, was significantly reduced in the DDT-30–40nt sperm fraction group compared to controls (Extended data Fig.4e–f). We also observed a significant reduction in glycogen levels and labyrinth fetal vascularization in DDT-30–40nt group placentas compared to controls (Extended data Fig.4g–j). However, unlike placentas in the DDT total sperm RNA group, we also detected a significant increase in the labyrinth area in placentas of the DDT-30–40nt sperm fraction group suggesting a compensation mechanism for the reduced placenta vascularization (Extended Figure 5a–c). While less pronounced, the DDT-15–25nt sperm fraction also impacted placental and fetal development (Extended data Fig.4k–r). Though there were no differences in fetal growth between the 15–25nt sperm fraction groups (Extended data Fig.4k–l), placentas in the DDT-15–25nt group weighed significantly less than controls (Extended data Fig. 4m–n). Placenta glycogen levels (Extended data Fig. 4o–p), but not fetal vascularization, were reduced in the DDT-15–25nt sperm fraction group (Extended data Fig.4q–r) compared to controls, while no major changes in placenta morphology were observed between groups (Extended Fig. 5d–f).

Overall, our data indicates that the entire DDT-induced sperm RNA load is required to drive the full placenta and metabolic phenotypes observed in DDT progeny. This aligns with our observations showing that DDT exposure significantly alters the levels of miRNAs, tsRNAs and rsRNAs as well as abundance of specific tsRNAs and rsRNAs fragments in sperm (Fig.2 a–i)

### Sperm RNAs from obese males increased placental efficiency and fetal growth

**Extended data Fig.6.**
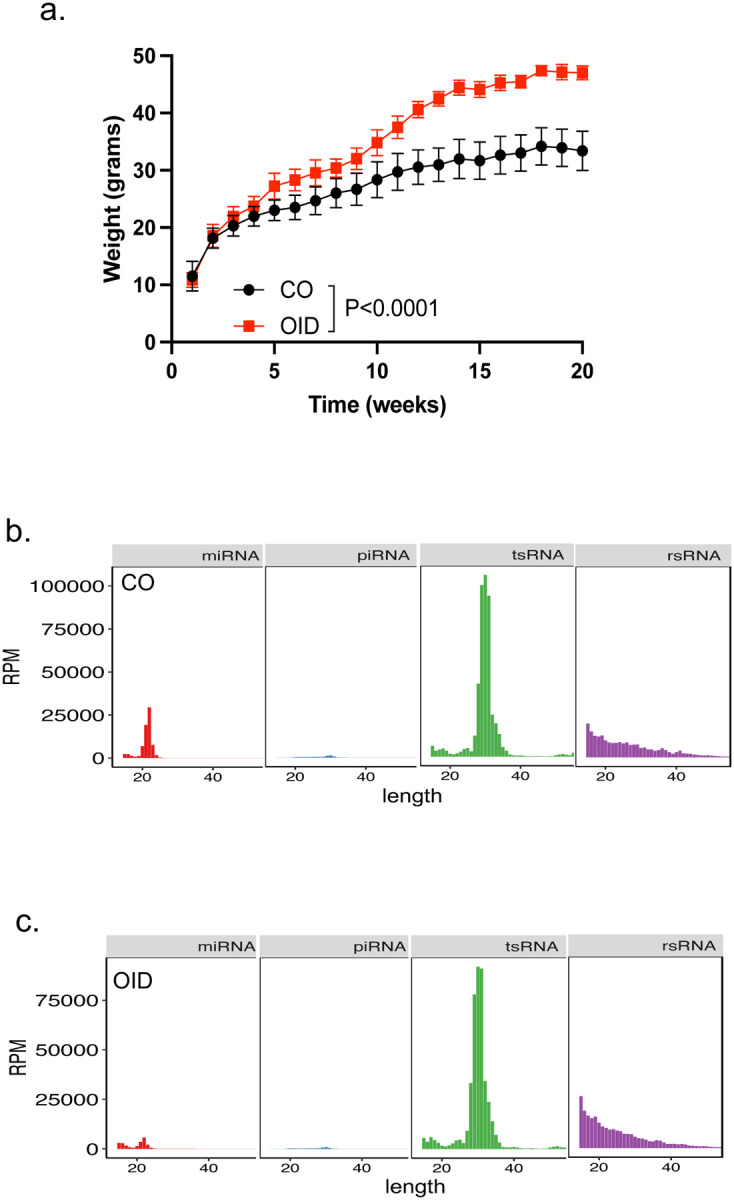
Males were fed an obesity-inducing diet (OID) or a control diet (CO) for 20 weeks. (a) Longitudinal bodyweight of CO and OID males. Data shown as mean–SEM, n=9–10 males/group. Data was analyzed by two-way ANOVA. (b-c) Read count (RPM) summary and length distributions of different sncRNA subtypes in sperm RNA-seq analysis of (b) CO or (c) OID-fed male mice (n=5–6/group).

**Extended data Fig.7.**
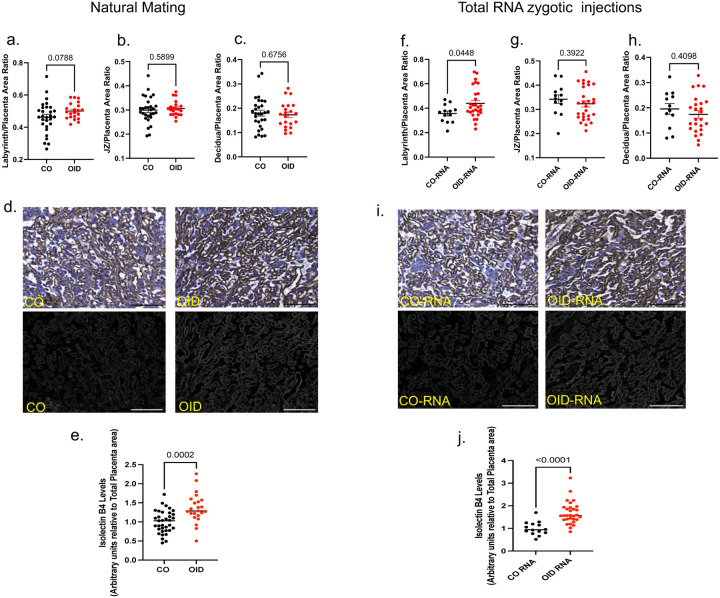
(a-c) Lean (CO) and obese (OID) male mice were mated to unexposed females or used for sperm extraction. Pregnant dams were euthanized on E13.5 for placenta and fetal collection. Layer-specific placenta surface area normalized to total placenta area and shown by study and group: (a) Labyrinth zone, (b) Junctional zone (JZ) and (c) Decidua. Placenta images were analyzed via image J software. Data shown as mean–SEM (horizontal bars in scatter plots, n = 21–29, 3–6 litters) (d) Representative pictures of placenta labyrinth zone showing IB4 staining (brown, fetal vessel marker, bar=100um). (e) Placenta fetal capillary quantification (IB4, normalized by total placenta area, n=23–35, 3–6 litters). (f-h) Naïve mouse zygotes were injected with the entire RNA load from the sperm of either CO or OID males. Injected zygotes were transferred into recipient female mice to produce offspring. Pregnant dams were euthanized on E13.5 for placenta and fetal collection. Layer-specific placenta surface area normalized to total placenta area and shown by study and group: (f) Labyrinth zone, (g) Junctional zone (JZ) and (h) Decidua. Placenta images were analyzed via image J software. Data shown as mean–SEM (horizontal bars in scatter plots, n = 12–28, 4–6 litters/group). (i) Representative pictures of placenta labyrinth zone showing IB4 staining (brown, fetal vessel marker, bar=100um). (j) Placenta fetal capillary quantification (IB4, normalized by total placenta area, n=14–30, 4–6 litters). All data were analyzed by t-test.

**Fig 3.**
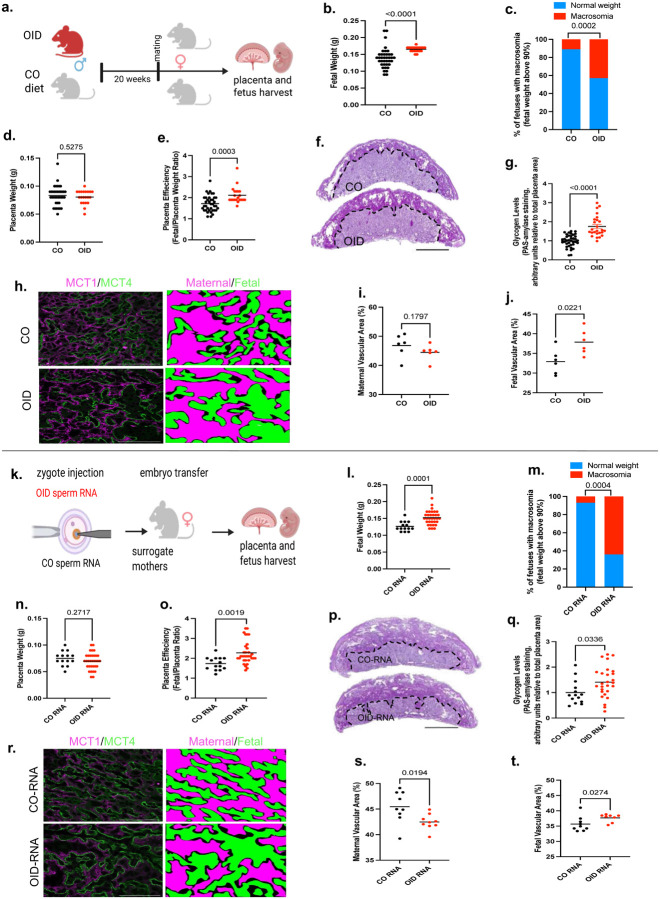
Sperm RNAs from obese males increase placenta efficiency and fetal growth (a) Experimental design: Lean (CO) and obese (OID) male mice were mated to unexposed females. Pregnant dams were euthanized on E13.5 for placenta and fetal collection. (b) Fetal weight (grams, n=26–40, 3–6 litters). (c) Percentage of fetal macrosomia (90th percentile fetal weight cut-off). (d) Placenta weight (grams, n=26–40, 3–6 litters). (e) Placenta efficiency (gram of fetus per gram of placenta, n=26–40, 3–6 litters). (f) Representative pictures of PAS (dark magenta, glycogen marker, bar=0.8mm) stained mouse placentas. (g) Placenta glycogen level quantification (PAS-amylase staining, normalized by total placenta area, n=25–38, 3–6 litters). (h) Representative picture of placental labyrinth zone stained for MCT1 (magenta, syncytiotrophoblast-I) and MCT4 (green, syncytiotrophoblast-II), demarcating the maternal blood sinusoids and fetal vessels, respectively (bar=50um). (i-j) Quantification of maternal(i) and fetal maternal(j) blood spaces (n=6, 3 litters). (k) Experimental design: Naïve mouse embryos at the zygote stage were injected with the entire RNA load from the sperm of CO or OID males. Injected zygotes were transferred into recipient female mice to produce offspring. Pregnant dams were euthanized on E13.5 for placenta and fetal collection. (l) Fetal weight (grams 4–6 litters, 14–33). (m) Percentage of fetal macrosomia (90th percentile fetal weight cut-off of the control group). (n) Placenta weight (grams, 4–6 litters, 14–33). (o) Placenta efficiency (gram of fetus per gram of placenta, 4–6 litters, 14–33). (p) Representative pictures of PAS (dark magenta, glycogen marker, bar=0.8mm) stained mouse placentas. (q) Placenta glycogen level quantification (PAS-amylase staining, normalized by total placenta area, n=14–27, 4–6 litters). (r) Representative picture of placental labyrinth zone stained for MCT1 (magenta, syncytiotrophoblast-I) and MCT4 (green, syncytiotrophoblast-II), demarcating the maternal blood sinusoids and fetal vessels, respectively (bar=50um). (s-t) Quantification of maternal (s) and fetal maternal (t) blood spaces (n=8–9, 4–5 litters). Data shown as mean (horizontal bars in scatter plots in b,d,e,g,i,j,l,n,o,q,s,t) or percentage (c,m). Data were analyzed by t-test or Mann-Whitney test (b,d,e,g,i,j,l,n,o,q,s,t) or Fisher’s exact test (c,m).

To further confirm the role of sperm RNAs in placentation, we employed the widely explored paternal obesity paradigm of epigenetic inheritance. Paternal obesity has been shown to alter the sperm RNA cargo in humans and animal models^2,10,12,45^ and to promote disease in offspring, including metabolic dysfunction and cancer.^2,45,46^ In rodents, paternal obesity is associated with alterations in placenta^47^ and birth weight.^46^ Population studies link high pre-conception paternal body mass index to abnormal birth weight, with both small for gestational age and macrosomia outcomes reported.^48,49^ Consistent with that, we found that mating of healthy weight female mice with obese males (fed an obesity-inducing diet [OID] for 20 weeks, Extended data Fig. 6a) led to greater placenta efficiency and increased fetal growth (Fig.3a–e) compared to controls. These phenotypes were linked to increased glycogens levels and enhanced labyrinth vascularization with expanded fetal vasculature in E13.5 placentas of OID offspring (Fig.3 f–j, Extended Fig. 7a–e).

As observed in the paternal DDT paradigm, sperm RNA zygotic injection recapitulated the original phenotype observed in progeny of OID sires, with increased fetal weights and placenta efficiency (Fig.3k–o). Further strengthening the notion that sperm RNAs modulate placenta development, we also observed an increase in glycogen levels in placenta of OID-RNA group compared to CO-RNA group (Fig.3p–q). OID-RNA zygotic injection also resulted in larger labyrinth area with extensive branching, increased fetal vascularization and reduced maternal sinuses compared to CO-RNA placentas (Fig.3r–t, Extended Fig. 7f–j).

These results indicate that obesity-driven sperm RNAs influence placenta function by increasing nutrient producing cells and expanding labyrinth vascularization that exceeds energy needs of the fetus, resulting in increased fetal growth. Our findings are also in line with prior reports^2,11^ mechanistically linking the sperm RNA load of obese males to the progeny’s disease phenotypes.

**Fig 4.**
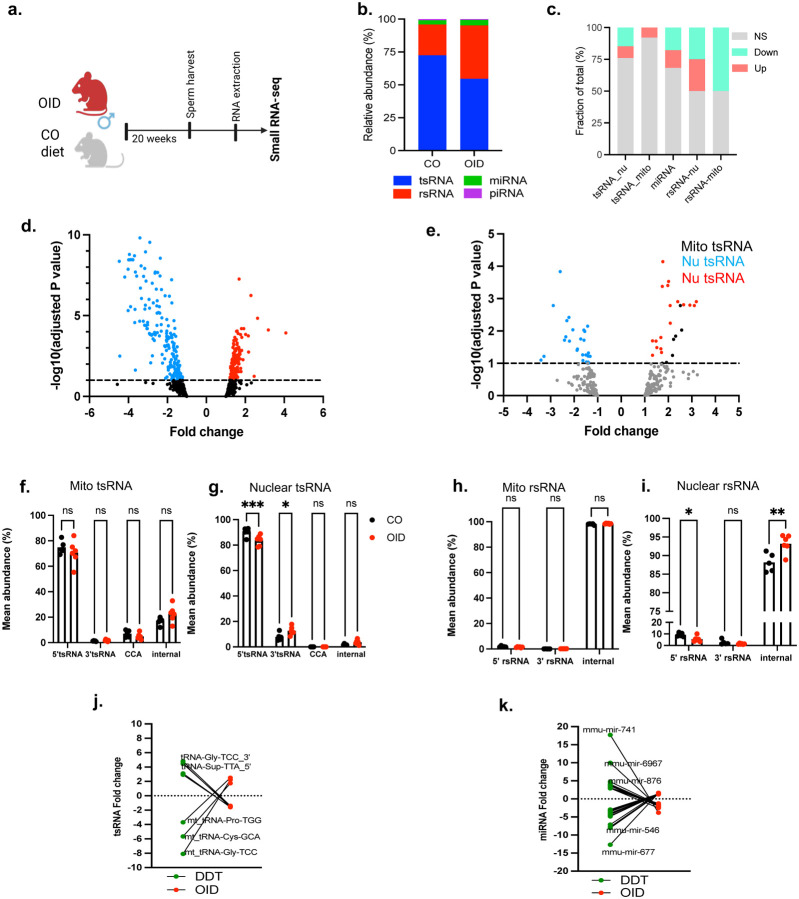
Pre-conception paternal obesity modulates the sperm non-coding RNA content. (a) Experimental Design: Lean (CO) and obese (OID) were used for sperm harvest. Sperm was used for RNA extraction and for small RNA-seq analysis. (b) Relative distribution of sncRNA subtypes in cauda spermatozoa from CO and OID male mice assessed via small RNA-seq (miRNA: microRNA: tsRNA; tRNA-derived small RNAs: rsRNA, rRNA-derived small RNAs; piRNA: piwi RNA). c) Subtype-specific fraction (percentage of the total reads) showing statistically significant up- or down-regulated cauda sperm sncRNAs. (d-e) Volcano plot representation of differentially expressed miRNAs, mitochondrial (mito) and nuclear tsRNAs. (f-i) Fragmentation patterns of mito and nuclear (f-g) tsRNAs or (h-i) rsRNAs in cauda sperm from CO and OID fed mice. Significance tested by a two-way ANOVA, followed by Holm-Sidak’s *t*-test (mean, *n* = 5–6; *P < 0.05, ***P < 0.001). (j-k) Differentially expressed tsRNAs fragments (j) or miRNAs (k) with opposite expression in sperm from DDT-treated or OID-fed males with specific examples labelled.

Small RNA-seq analysis of the sperm from obese (OID) and lean control mice (Fig. 4a–i, Extended data Fig. 6b–c) detected differential expression in rsRNAs, tsRNAs and miRNAs, but not piRNAs, between groups (Fig. 4c–e). Further analysis of tRNA fragmentation patterns revealed a significant decrease in 5’-end nuclear tsRNAs and higher abundance in 3’-end nuclear -derived tRNA fragments in sperm of obese males compared to controls (Fig. 4f–g). The abundance of nucleus-derived, but not mitochondrial-derived, 5’-end rsRNA fragments was significantly decreased while internal rsRNA fragments were significantly increased, respectively, in sperm of OID males (Fig. 4h–i).

Because zygotic injection of sperm RNA of DDT and obese males led to opposite fetal and placenta phenotypes, we next examined whether there were overlapping differentially expressed sperm sncRNAs but with opposite expression in those groups. Indeed, 8 tsRNAs (5 nuclear and 3 mitochondrial) and 25 miRNAs showed opposite expression in sperm of DDT-exposed males compared to obese males (Fig. 4j–k, Supplementary table 1), suggesting these sncRNAs could play a role in embryonic cell specification and placentation.

### Environmentally-induced sperm RNAs shape pre-implantation embryo development and cell lineage specification

**Fig 5.**
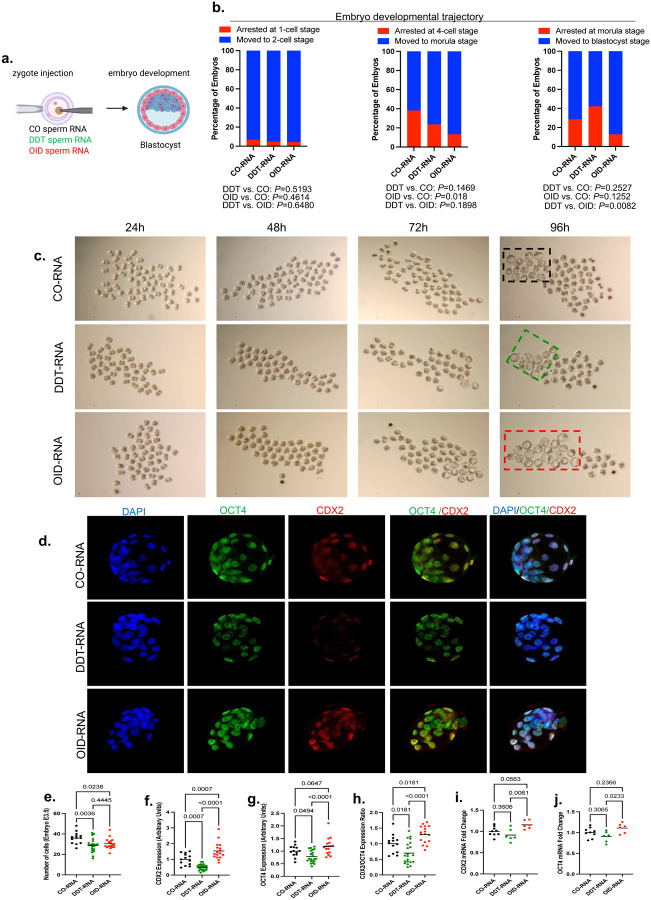
Environmentally-induced sperm RNAs alters pre-implantation embryonic developmental trajectory and stem cell specification. (a) Experimental design: Naïve mouse zygotes were injected with the entire RNA load from the sperm of either CO, DDT-exposed or obese (OID) males. Injected zygotes were cultured for up to 96 h and developmental trajectory assessed every 24h until the blastocyst stage. Harvested blastocysts were stained with antibodies against CDX-2 or OCT-4 and counterstained with DAPI and imaged (63x). (b) Developmental trajectory of pre-implantation embryos injected with sperm RNA from CO, DDT-exposed or OID males (*n*=42–60 injected embryos). (c) Representative images showing CO-RNA, DDT-RNA and OID-RNA injected embryos at different stages (2–4 cell stage, morula and blastocyst) assessed at 24, 48, 72 and 96 h post-injection. (d) Representative pictures of blastocyst stage embryos showing CDX-2 (red) or OCT-4 (green) protein expression. (e-g) Quantification of total number of cells (e), CDX-2 (f) or OCT4 (g) protein expression levels in blastocyst-stage embryos (*n* = 13–21) injected with sperm RNA from CO, DDT-exposed or OID males. (h) Ratio of CDX-2/OCT4 expression in the same embryos. (i-j) qPCR quantification of mRNA levels for *Cdx-2* and *Oct4* in blastocysts (*n* = 6–9 pooled embryo samples) from CO, DDT-RNA or OID-RNA groups. Significance tested by Fisher’s exact test (b) or one-way ANOVA (e-j), followed by Holm-Sidak’s test. Data shown as percentage (b) or mean (horizontal bars in scatter plots in e-j).

The process of placentation starts in pre-implantation development when embryonic cells undergo their first cell fate decision to differentiate into either the inner cell mass (ICM) or the trophectoderm (TE) cell lineages that will give rise to the embryo or placenta, respectively.^16^ To assess the impact of zygotic injection of sperm RNA from either DDT-exposed or OID-fed males on pre-implantation embryonic development, we performed in vitro embryo culture and monitored development for 96h (Fig.5a). Harvested blastocysts were then stained with antibodies against OCT-4 and CDX-2, to identify the ICM and TE cell lineages, respectively.

Embryos injected with sperm RNA from OID-fed males showed accelerated development, with fewer embryos arrested at any stage and more embryos reaching the morula or blastocyst stage compared to those injected with sperm RNA from CO or DDT-exposed males, respectively. Interestingly, while embryos injected with sperm RNA from DDT-exposed males did not show altered development until the morula stage, a smaller percentage of morula-stage embryos reached the blastocyst stage compared to the OID group (Fig.5b–c).

Changes in embryo development were accompanied by alterations in both total number of cells and stem cell specification (Fig.5d–j). Embryos injected with DDT sperm RNA showed reduction in overall number of cells (Fig.5e), protein expression levels of CDX-2 and OCT4 as well as CDX-2/OCT4 ratio compared to both the control and OID-RNA groups (Fig.5f–h). Though blastocysts in the OID-RNA group also displayed a smaller number of cells compared to controls, these embryos showed higher expression of CDX-2 and OCT4 as well as CDX-2/OCT4 ratio compared to both the CO-RNA and DDT-RNA groups (Fig.5e–h). The CDX-2 and OCT4 protein level results were largely confirmed at the transcriptional level by qPCR (Fig.5i–j).

Altogether, our data suggest that environmentally-induced RNAs impact both development and cell lineage specification in pre-implantation embryos which may ultimately influence embryo implantation capacity and the placentation process. An optimal level of CDX-2 is required for adequate differentiation and segregation of the ICM and TE cell lineages and lack of CDX-2 expression in the early embryo leads to hatching and implantation failures.^50,51^

### Environmentally-induced sperm RNAs alter the mature placenta cell population and imprinted gene landscape

**Fig. 6.**
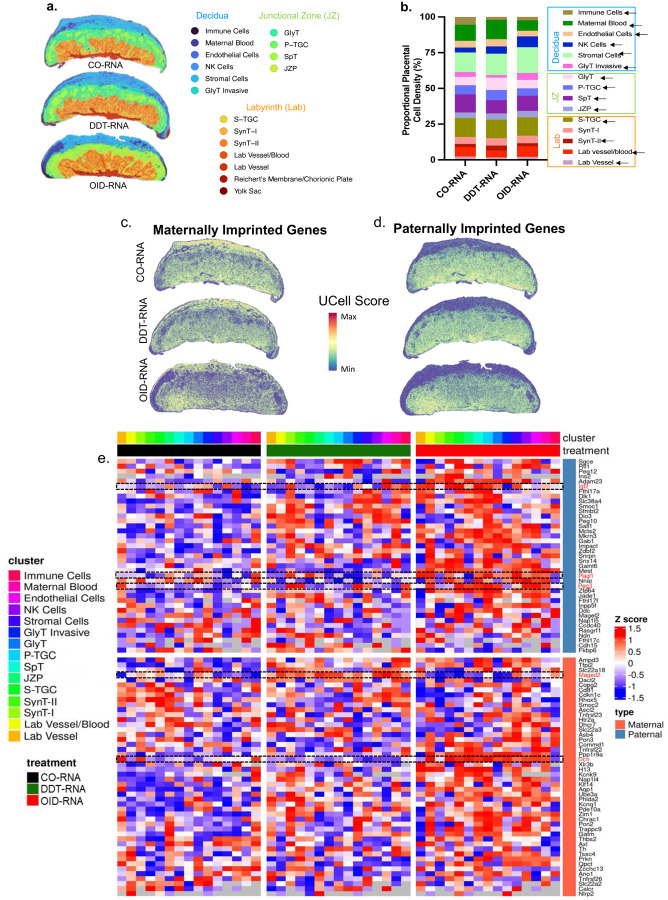
Environmentally-induced sperm RNAs alter density of placental cell populations and the imprinted gene landscape. (a) Representative sections of CO-RNA, DDT-RNA, or OID-RNA mouse placenta (n=4/group) harvested at E13.5 for ST (Illumina-Visium HD platform), showing with 17 annotated clusters (*NK*, Natural killer cells; *GlyT*, glycogen trophoblast; *SpT*, spongiotrophoblast; *P-TGC*, Parietal trophoblast giant cell; *JZP*, Junctional zone precursor; *SynT*, syncytiotrophoblasts; *S-TGC*, Sinusoidal trophoblast giant cell) (b) Placenta cell composition by group: Proportional density (%) of cells normalized by the respective placenta zone area (Reichert’s membrane/chorionic plate and yolk sac were excluded); Arrows depict cell clusters with significant differences (Chi-squared test, p<0.05, additional details in Supplementary table 2) in cell density between groups. (c-d) Spatial expression of imprinted genes in the placenta of the CO-RNA, DDT-RNA, or OID-RNA groups. Ucell scores of all (c) maternally and (d) paternally expressed imprinted genes captured by spatial transcriptomics within the imprinted gene set are visualized spatially in different placenta regions. (e) Heatmap showing expression of imprinted genes in placentas of the CO-RNA, DDT-RNA, or OID-RNA groups. Genes highlighted in red font and marked by dotted lines were selected for validation.

To further characterize cellular and transcriptional alterations underlying placental dysfunction linked to environmentally-induced sperm RNAs, we undertook a spatial transcriptome (ST) analysis of E13.5 placentas in the CO-RNA, DDT-RNA and OID-RNA groups. We identified the main placental cell populations in the decidua, junctional zone and labyrinth layers as previously described^52^, with a total of 17 major clusters detected (Fig 6a). ST analysis uncovered group-dependent alterations in cell proportion within specific placenta clusters (Fig 6b, Supplementary table 2). For instance, while the DDT-RNA placenta group showed significantly higher proportional density of GlyT, it had lower density of invasive GlyT compared to placentas in the CO-RNA and OID-RNA groups. In contrast, the OID-RNA placenta group showed significantly higher density of invasive GlyT compared to both the control and DDT-RNA groups. This is consistent with the low and high placental glycogen levels detected in the DDT-RNA and OID-RNA groups, respectively (Fig.1 p–q, Fig.3 p–q).

In agreement with the placenta vasculature deficiency in the DDT-RNA group (Fig.1r–t, Extended data Fig. 2i–j), the ST analysis revealed a significant reduction in cell density in the lab vessel/blood cluster in this group compared to the CO-RNA and OID-RNA groups. Conversely, the OID-RNA placenta group showed significantly higher proportional density of stromal and NK cells compared to both the CO-RNA and DDT-RNA groups (Fig 6b, Supplementary table 2).

Imprinted genes play a critical role in placentation^16,22,27^ and control fetal growth and development by regulating the nutrient supply from mother to fetus by managing placental nutrient transport and vascularization. They also provide a mechanism that balances resource allocation, with paternally expressed genes often promoting fetal growth and maternally expressed genes limiting it. In line with previous reports^52^, the expression of imprinted genes was spatially compartmentalized based on parent-of-origin (Fig.6c–d), with paternally imprinted genes enriched in the junctional zone and labyrinth. In the group comparison (Fig.6e), both paternally and maternally imprinted genes showed overall higher expression in the OID-RNA placenta group compared to the other groups, suggesting activation of a resource allowance process.

**Extended data Fig. 8.**
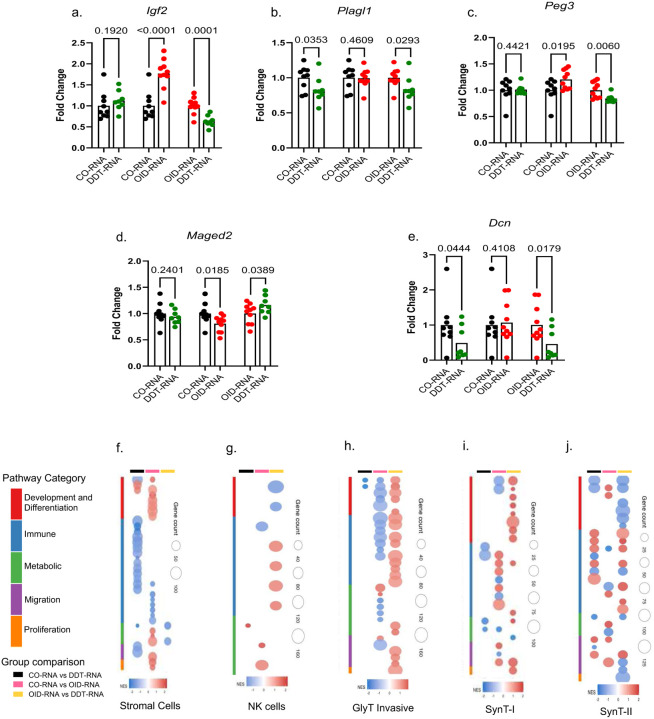
(a-e) qPCR validation of the expression of paternally and maternally imprinted genes in placentas of the CO-RNA, DDT-RNA, or OID-RNA groups (n=8–10/group). Significance tested by t-test. Data shown as mean (horizontal bars in scatter plots in a-e). (f-j) Top biological processes enriched in selected cell clusters in spatial transcriptome data of placentas of the CO-RNA, DDT-RNA, or OID-RNA groups (n=4/group).

There were also cell cluster specific alterations. For instance, the paternally expressed genes *Igf2*, *Plagl1* and *Peg3* were elevated in cell clusters of the junctional zone, labyrinth or the decidua in OID-RNA placentas compared to the DDT-RNA or CO-RNA groups. These imprinted genes play a crucial role in placenta morphogenesis, labyrinth vascularization and trophoblast cell numbers.^22,52,53^ *Maged2*, a maternally expressed gene, involved in oxygen fluctuation response^54^, was highly expressed in the majority of clusters in the DDT-RNA placentas compared to both OID-RNA and CO-RNA groups. *Dcn*, another maternally expressed gene that regulates decidualization^55^, was reduced in DDT-RNA placentas compared to the OID-RNA group and, to a less extent, the CO-RNA group. q-PCR analysis largely validated these group-specific alterations in placenta expression of *Igf2*, *Plagl1 Peg3, Maged2* and *Dcn* (Extended data Fig 8a–e).

In addition to changes in imprinted genes, zygotic injection of sperm RNAs from DDT-exposed or OID-fed males caused other cell-type specific transcriptional effects compared to controls (Supplementary table 3). Functional enrichment analysis showed an over-representation of GO terms associated with development and differentiation, immune response, metabolic, migration and proliferation processes. Activation or suppression of these pathways were cluster and group dependent (Extended data Fig 8f–j, Supplementary table 4). For instance, stromal cells and SynT-II showed an increase in development and differentiation pathways in OID-RNA compared with the CO-RNA group (Extended data Fig 8f,i). In contrast, these same pathways were decreased in SynT-II of the DDT-RNA placentas compared to the OID-RNA group (Extended data Fig 8j). Immune pathways were down-regulated in stromal cells of DDT-RNA placentas compared to controls (Extended data Fig 8j) but increased in NK cells, invasive GlyT and SynT-II in the DDT-RNA group compared to the OID-RNA group (Extended data Fig 8g,h,j). Metabolic, migration and proliferation pathways were activated in invasive GlyT in DDT-RNA placentas compared to the OID-RNA group (Extended data Fig 8h) and in stromal cells of OID-RNA placentas compared to controls (Extended data Fig 8f).

Our data support a role for environmentally-induced sperm RNAs in shaping placenta cellular composition and function through transcriptional regulation of imprinted genes and pathways controlling development and differentiation, metabolism, immunity, cell proliferation and migration in specific cell clusters.

## Discussion

**Extended data Fig.9.**
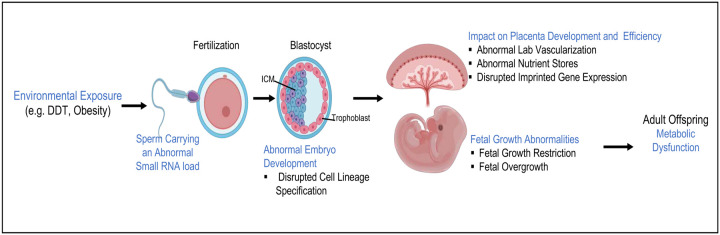
Potential mechanism by which pre-conception paternal experiences and environmentally-induced sperm RNAs modulate placentation and impact progeny development and traits.

Sperm RNAs are sensitive to the environment and have been shown to mediate paternally-induced epigenetic inheritance of traits.^2,5–7^ Despite the evidence for their role in heritable non-genetic phenotypical variation, the precise mechanisms by which sperm RNAs alter the course of embryonic development to shape the progeny’s traits remains poorly understood. Using two different mouse models of male pre-conception experiences, this work shows that environmentally-induced sperm RNAs shape placenta development and function with direct consequences for fetal growth and disease outcomes in the progeny (Extended data Fig.9).

Previous observational studies report that paternal factors are associated with placenta function in both human and rodent models.^28–34,48,49^ Our data extend those findings and provides the first direct evidence that the sperm RNA cargo regulates placentation. Our results show that environmentally-driven sperm RNAs shape placenta cellular composition, nutrient stores and vascular branching. The by-product of these sperm-induced placenta events is either increased or reduced placenta efficiency leading to fetal growth restriction, as observed in the paternal DDT model, or fetal overgrowth, as detected in the paternal obesity paradigm.

In eutherian mammals, the inner cell mass and the trophectoderm are the first definitive embryonic cell lineages to appear^21^. The integrity and function of the trophectoderm is critical for blastocyst implantation. The transcription factors CDX-2 and OCT4 control cell lineage segregation to the trophectoderm or ICM, respectively. Embryos lacking CDX-2 expression fail to properly hatch or implant.^50,51^ Our data indicate that environmentally-induced sperm RNAs interfere with the process of cell fate decision. Injection of the sperm RNA load from DDT-treated males into zygotes decreased the CDX-2/OCT4 expression ratio in blastocysts, while zygotic injection of sperm RNAs from obese males increased this ratio. The end result is altered embryo quality, with either slow (DDT-RNA group) or accelerated embryo development (OID-RNA group), which could influence implantation competence and the placentation process. In support of these results, it was recently reported that sperm sncRNAs were predictors of embryo quality in humans.^56^ Consistent with the inverse effects of DDT or obesity-induced sperm RNAs on embryo development and placenta phenotypes, we also observed overlapping, but opposite, differential expression of a specific subset of sperm sncRNAs in the two groups, indicating that they could be good candidates for further investigation.

We found that sperm RNA-driven placenta phenotypic changes are related to alterations in imprinted genes. Genomic imprinting plays a critical role in placentation^16,22,27^ and controls fetal growth by regulating the placental supplies of energy to the fetus through both nutrient transport and vascularization. From an evolutionary perspective, imprinted genes provide a mechanism that balances resource allocation during development, with paternally expressed genes promoting fetal growth and increasing metabolic demands on the mother, while maternally expressed genes limit resource availability to the fetus. In healthy pregnancies, these maternal-offspring interactions are of mutual cooperation to ensure both maternal and fetal well-being. Our findings indicate that environmentally-induced sperm RNAs could exacerbate this genetically-driven conflict by disrupting imprinted gene expression. In the OID-RNA group, we observed increased placental expression of both maternally and paternally imprinted genes (including *Igf2*), suggesting that the increased fetal growth and metabolic demands trigger a response to preserve maternal resources.

In line with the reduced placenta efficiency, placenta expression of paternally expressed genes including *Igf2*, *Plagl1* and *Peg3* was decreased in the DDT-RNA group. Previous work showed that *Plagl1* is a master regulator of the imprinted gene network^52^ and, along with *Igf2*, drives the expansion of the feto-placental microvasculature to align with fetal needs^22^. *Peg3* has been reported to regulate trophoblast proliferation and hormone levels^53^. Interestingly, *Maged2*, a maternally expressed gene required for cAMP/PKA pathway activation under hypoxic conditions^54^, was highly expressed in DDT-RNA placentas, signaling an attempt to facilitate cell adaptation and survival under a low oxygen environment. *Dcn*, another maternally expressed gene, controls trophoblast invasion and its suppression in human placenta is linked to fetal growth restriction^55^.

Our data is also consistent with evidence showing a role for human imprinting disorders in fetal growth. For instance, IGF2 dysregulation^57^ in Beckwith-Wiedemann Syndrome (*IGF2* over-expression) and Silver-Russell Syndrome (*IGF2* under-expression) leads to fetal overgrowth and fetal growth restriction syndromes, respectively. Mutations in *MAGED2*, a regulator of sodium transporters in the placenta and fetal kidney, are behind Bartter's syndrome which is characterized by extreme prematurity.^58^ Data from mouse models also support our findings and show that placental KO of *Igf2* increases fetal growth restriction due to low fetal glycemia^22^. Likewise, placenta *Peg3* KO leads to placenta dysfunction with reduced spongiotrophoblast and glycogen cell lineages along with diminished glycogen stores^53^.

Abnormal placenta function and adverse events in fetal development have been associated with disease later in life^21,22^ and our findings offer a potential mechanism by which paternal experiences modulate risk of chronic diseases in the progeny reported in humans^4,10,59–64^ and animal models.^65,66^ Although our study does not provide direct evidence that the observed placenta abnormalities are responsible, we show that sperm RNA of DDT-treated males transfer propensity to metabolic dysfunction to the progeny. Our findings are also consistent with prior studies^2,11^ mechanistically linking the sperm RNA load of obese males to the progeny’s disease phenotypes.

To the best of our knowledge, this work provides the first experimental evidence for a direct role of sperm RNAs in placentation with implications for fetal growth and disease risk in offspring later in life. While our study offers conceptual advances that can help explain how epigenetic inheritance of traits through the male germline occurs, we acknowledge some limitations. Although our data suggest that more than one subtype of sperm sncRNAs is implicated in regulating placentation, what specific role they play needs to be further elucidated. It will be especially important to examine how sperm RNAs impact stem cell specification in pre-implantation embryos, and whether they directly or indirectly regulate imprinted genes which are critical for fetal and placental development. Moreover, longitudinal studies will be required to comprehensively explore the spatiotemporal impact of environmentally-induced sperm RNAs on the placentation process (starting at implantation and throughout key placentation stages) to fully understand the cellular and molecular underpinnings. Prospective studies to show a relationship between sperm RNA and placenta function in human pregnancies will also be critical. We also we acknowledge that, in addition to sncRNAs, it is possible that long coding RNAs^67^ and other sperm epigenetic carriers^68–70^ may play a role in placenta development.

We speculate that regulation of placentation by environmentally-induced sperm RNAs disturbs the development of fetal organs, particularly tissues that are sensitive to oxygen and nutrients supply^16^ (e.g. brain, heart, pancreas), and additional studies are required to thoroughly evaluate the tissue-specific adverse consequences. There is also a relationship between placenta function and maternal physiology in pregnancy.^71,72^ The association between the content of sperm-RNAs at conception with maternal health during gestation and their role in pregnancy-related diseases also remains to be explored. Interestingly, a recent study suggests that the fetus manipulates maternal metabolism through placental *Igf2* and that its deficiency impairs normal maternal adaptation in pregnancy.^22^

In conclusion, although the mechanistic details await further experimentation and confirmation in humans is needed, our findings could have significant public health implications. This study adds to the evidence suggesting that, in addition to maternal pre-natal factors, male pre-conception experiences and health need to be considered to ensure optimal pregnancy outcomes. Evolutionarily, our findings offer a novel non-genetic mechanism by which fathers could shape resource allocation to their progeny via sperm RNAs.

## Materials and Methods

### DDT Exposure Model

#### DDT exposure:

The C57BL/6NTac strain of mice (Taconic Biosciences) was used in this experiment. Adult male mice (8–10 weeks of age) were either treated daily with a DDT solution (1.7 mg/kg) or vehicle-control solution (vegetable oil) via oral gavage for 17 days. Duration of exposure was chosen in order to encompass the entire period of post-testicular sperm transit in the epididymis (which takes about 15 days in mammals), the last stage of sperm maturation^73^. The DDT solution used mimics the formulation of DDT before its ban in the U.S.: 77.2% p,p’-DDT (AccuStandard, catalog #P-029N) and 22.8% o,p’-DDT (AccuStandard, catalog #P-028N) as described before^74^.

At the end of the exposure period, DDT or vehicle-control exposed male mice were mated overnight to unexposed females or used for mature sperm harvest.

All animal procedures were approved by the Georgetown University Animal Care and Use Committee, and the experiments were performed following the National Institutes of Health guidelines for the proper and humane use of animals in biomedical research. Animals were randomized to each exposure group and treatments were performed blindly.

#### Measurement of DDT metabolites:

Liver tissues from CO and DDT-treated males were pooled (5 samples/group, n=2/group) and used to determine the levels of DDT’s main metabolites. Measurements were performed commercially by Pacific Rim Labs, a fully accredited laboratory providing analysis of DDT and other persistent organic pollutants. Briefly, samples were fortified with each of the six 13C-DDT isotopes and then extracted with hexane. The extract was columned on Florisil and analyzed by GC-MS/MS. The instrument was calibrated with a five-point calibration covering the range of 0.2–400 ng/g or mL.

#### Western blot:

Total protein lysates were prepared from epididymis and testis of CO or DDT-treated males using RIPA buffer (Thermo Fisher Scientific, catalog #89901) supplemented with Halt^™^ Protease Inhibitor Cocktail (Thermo Fisher Scientific catalog #78429). Equal amounts of protein were resolved on 4–12% SDS-polyacrylamide gels (Invitrogen, #NP0321) and transferred using the iBlot^®^ 7-Minute Blotting System (Invitrogen, catalog #IB23001). Membranes were blocked with 5% nonfat dry milk for 1 h at room temperature and incubated overnight at 4 °C with anti-Androgen Receptor (AR) primary antibody (Proteintech, catalog #22089-I-AP). After washing, membranes were incubated with HRP-conjugated secondary antibody (Proteintech, catalog #SA00001–4) for 1 h at room temperature. Signals were developed using a chemiluminescent HRP substrate (Advansta, catalog #K-12049-D50) and imaged with an Amersham^™^ Imager 600 (GE Healthcare). Band intensities were quantified using Image J Fiji and normalized to β-actin (Cell Signaling Technology, catalog #8457).

#### Testosterone levels:

Plasma testosterone levels in CO or DDT-treated males were measured using commercially available ELISA kit (CrystalChem, catalog #80552), according to manufacturers’ instructions.

#### Breeding and timed placenta harvest:

Non-treated female mice were mated with either DDT or vehicle-control exposed males overnight. Mating was confirmed by detection of a vaginal plug. This was considered the first day of pregnancy or embryonic day (E) 0.5. Pregnant dams were housed in groups of two with free access to food and water and euthanized on E13.5, after placentation is complete in mice^17^. Placentas and fetuses were weighed upon dissection and either snap frozen or fixed in 10% neutral-buffered formalin. DNA from fetal tail tips were used for sex determination via commercial genotyping (Transnetyx, Inc.) of the Y chromosome specific gene, SRY.

#### Placenta efficiency and morphometric assessments:

Placenta efficiency was estimated by dividing fetal weight in grams by their respective placenta weight also in grams.

Placenta morphometry was evaluated in two mid-sagittal serial placenta sections per sample using the Image J Fiji image processing software. Morphometric ends points were total placental surface area and surface area of each placental layer (labyrinth, junctional zone and maternal decidua). Briefly, an image outline of each individual mid-sagittal serial placenta section (PAS staining) was traced using the Image J software tracing function. The number of pixels in the selected area was then calculated by the software. These procedures were then repeated to determine the area of each specific placenta layer.

Glycogen (GlyT) cells within the junctional zone were stained with periodic acid–Schiff (PAS), followed by digestion without and with α-amylase, using a commercial PAS staining system (Sigma-Aldrich, catalog #395), according to manufacturer’s instructions. Fetal capillaries in the labyrinth zone were detected via staining for isolectin B4, a marker for endothelial cells (Sigma-Aldrich, catalog #L5391)

#### Mature spermatozoa collection and purification:

For sperm harvest, male mice were euthanized and caudal epididymis dissected. The epididymis was collected, punctured, and transferred to a tissue culture dish containing M2 media (M2 Medium-with HEPES, without penicillin and streptomycin, liquid, sterile-filtered, suitable for mouse embryo, Sigma-Aldrich, M7167) where it was incubated for 1 hour at 37°C. Sperm samples were isolated and purified from somatic cells. Briefly, the samples were washed with PBS, and then incubated with SCLB (somatic cell lysis buffer, 0.1% SDS, 0.5% TX-100 in Diethylpyrocarbonate water) for 1 hour. SCLB was rinsed off with 2 washes of PBS and the somatic cell-free purified spermatozoa sample pelleted and used for RNA extraction using a Qiagen miRNeasy kit (Qiagen, catalog #217084).

#### Sperm RNA fraction purification:

Sperm RNA populations of different sizes were separated by electrophoresis using a denatured 15% polyacrylamide gel with 7M urea (Invitrogen, catalog #EC68855). The gel was stained with SYBR green I solution and sperm fractions containing 15–25nt or 30–40 nt fragments were excised (The 15–25nt fraction contains the majority of miRNAs but also harbors piRNAs and smaller tsRNAs and rsRNAs. On the other hand, the 30–40nt sperm RNA fraction is composed of mostly tsRNAs and rsRNAs, but may also contain some larger piRNAs). RNA fragments were eluted from gel using a 0.3 M NaCl-TE solution overnight with shaking. Samples were filtered, precipitated using isopropanol and resuspended. Fragment size was confirmed using the Agilent 2100 Bioanalyzer. Purified samples will be kept at −80°C until use.

#### Zygote RNA microinjections:

To induce super-ovulation, female mice (C57BL/6) received intra-peritoneal injections of 5 IU of pregnant mare’s serum gonadotropin (PMSG, LEX Life Sciences, catalog #A22721K). About 48 hours after the PMSG injection, mice were injected with 5 IU of human *chorionic gonadotropin* (hCG, Sigma-Aldrich, catalog #C1063). Super-ovulated females were mated with c57bl/6 male mice. About 16 hours later, fertilized eggs were harvested. Zygotes were injected with the entire RNA load or specific RNA fractions from sperm of CO and DDT-treated males. For zygotic injections using the total sperm RNA load, each embryo received 10 pL injection of the RNA solution at 2ng/μL (the amount of sperm RNA injected is the equivalent to RNA from 10 sperm cells^5,11,75^ or approximately 20 femtograms per injection). For zygotic injections of sperm RNA fractions, each embryo received 10 pL injection of the RNA solution at 2ng/μL of the 30–40nt sperm fraction or at 0.25ng/μL of the 15–25nt sperm fraction, based on the proportional abundance of each RNA fraction in sperm.

#### Embryonic transfer:

Sperm RNA-injected zygotes were transferred into recipient female mice to produce offspring as described before^7^. Briefly, recipient females were mated with vasectomized males overnight and identified by copulation plug before the zygote transfer. Each recipient female was implanted with 25–30 zygotes. Dams were housed in groups of two with free access to food and water. Pregnant dams were either allowed to deliver the offspring or euthanized on embryonic day (E)13.5 for placenta collection as described below. All mice utilized in this study were kept on a standard chow diet including the exposures and breeding periods, for the extent of pregnancy (21 days) and after birth. Pups were weaned on postnatal day (PND) 21 and used to study metabolic function in adulthood.

#### Assessment of metabolic parameters in offspring:

Glucose tolerance test (GTT) and insulin tolerance test (ITT) were performed in 20 to 24 weeks old mice. For the GTT, mice were fasted for 6 hours, injected i.p. with glucose (2.5g/kg, Sigma-Aldrich, catalog #G8769) and glucose measurements taken at 0, 15, 30, 60, and 90 min using a Accu-check meter. For the ITT, mice were fasted for 4 hours, injected i.p. insulin (0.75 U/kg, Lilly, catalog #00002-8215-01), glucose measurements at 0, 15, 30 and 45 min using an Accu-check meter. ITT values were normalized to baseline glucose levels before data analysis.

### Paternal Obesity Model

#### Dietary exposure:

The C57BL/6NTac strain of mice (Taconic Biosciences) was used in this experiment. Males were fed an Obesity-Inducing-Diet (OID, AIN93G-based diet 57.1% energy from fat, 5.2 Kcal/g, TD.160019, Teklad Diet) for 20 weeks. Another cohort of age-matched male mice were fed a control, nutrient-balanced diet (CO, 3.8 Kcal/g). Animals were randomized to each dietary group. Animal body weight was recorded weekly. At the end of the exposure period, obese (OID) males or lean controls were mated overnight to unexposed female mice to generate timed pregnancies for placenta and fetal harvest or used for mature sperm harvest.

#### Breeding and timed placenta harvest:

Healthy normal weight female mice were mated with obese (OID) or lean control (CO) males overnight. Mating was confirmed by detection of a vaginal plug. This was considered the first day of pregnancy or embryonic day (E) 0.5. Pregnant dams were housed in groups of two with free access to food and water and euthanized on E13.5. Placentas and fetuses were harvest, processed and analyzed as described for the Paternal DDT Exposure Model.

#### Mature spermatozoa collection and purification:

CO and OID-fed male mice were euthanized and caudal epididymis dissected for sperm was harvest which processed and used for RNA extraction as described for the Paternal DDT Exposure Model.

#### Zygote RNA microinjections:

To induce super-ovulation, female mice (c57bl/6) received intra-peritoneal injections of 5 IU of pregnant mare’s serum gonadotropin (PMSG). About 48 hours after the PMSG injection, mice were injected with 5 IU of human *chorionic gonadotropin* (hCG). Super-ovulated females were mated with *c57bl/6* male mice. About 16 hours later, fertilized eggs were harvested. Zygotes were injected with the RNA load from sperm of CO and OID-fed males. Each zygote received 10 pL injection of the sperm RNA load at 2ng/uL.

#### Embryonic transfers:

Sperm RNA-injected zygotes were transferred into recipient female mice to produce offspring as described above. Briefly, recipient females were mated with vasectomized males overnight and identified by copulation plug before the zygote transfer. Each recipient female was implanted with 25–30 zygotes. Dams were housed in groups of two with free access to food and water. Pregnant dams were euthanized on embryonic day (E)13.5 for tissue collection. Placentas and fetuses were harvested, processed and analyzed as described for the Paternal DDT Exposure Model.

### Experiments Performed Using Both Models

#### Sperm sncRNA Sequencing:

Total RNA from sperm of DDT or OID males was isolated from purified sperm using Qiagen’s miRNeasy extraction kit, according to the manufacturer’s instructions. RNA integrity and quality examined by Bioanalyzer 2100 (Agilent Technologies). Small non-coding RNA transcript libraries were constructed according to the QIAseq small RNA Library Kit (Qiagen). After quality control, the library preparations were sequenced on an Illumina platform and reads generated.

While this sncRNA detection method is effective in identifying all major sncRNA subtypes, the library preparation protocol may not fully capture highly modified sncRNAs (e.g. rsRNAs and tsRNAs).

Raw data quality was checked using FastQC (v0.11.9), and adapter trimming on raw data were performed using Cutadapt (v3.5). The adaptor sequence was trimmed using in-built Cutadapt pipeline and sequences with the length of 15–45 nucleotides were further processed. Reads with low quality (quality score < 25, error rate > 10%) or reduced length after trimming (<15 bp) were removed before alignment. Trimmed and quality-filtered reads were used for further analysis. Sequencing files were aligned and annotated to the mouse genome (mm10) using the SPORTS1.1 pipeline^76^ with default parameters and a maximum number of mismatches of 2. Reference genome and small RNA annotation databases were downloaded from the SPORTS website. This included the mm10 genome files, miRNA from miRbase 21, rRNA from National Center for Biotechnology. Information (NCBI) Nucleotide, tRNA from GtRNAdb, piRNA from pirBase and piRNAbank, other ncRNA from ensembl (release-89), and rfam 12.3. The raw count tables generated by SPORTS were annotated to small RNA biotypes. Averages were aggregated across biotypes (rsRNA, tsRNA, miRNA and piRNA) using the default annotations in SPORTS result output files.

tRNA and rRNA derived small RNAs (tsRNAs and rsRNAs) reads identified by SPORTS were further annotated based on their mapping positions along the full-length reference sequences. RNA fragment end positions were calculated from the mapped start position and sequence length. tsRNAs and rsRNAs fragments were classified into positional categories: 5′ end, 3′ end (and 3′ end-CCA for tsRNAs) and internal as previously described.^76,77^ Raw counts for each tsRNAs and rsRNAs fragments and positional category were normalized to total clean reads and reported as reads per million (RPM). The fragments with at least 0.01 RPM in all of the samples and lengths between 16 and 45 nucleotides were retained for further analysis. Next, edgeR was used to identify differentially expressed fragments. All of the analyses were carried out using different packages in R version 4.1.2 and Bioconductor version 3.14. Fold change differences with an P adjusted value <0.1 were considered significant.

For the quantification of specific rsRNA or tsRNA fragment subtypes, the RPM values for each subtype were normalized to the total fragment count within the control and treated groups. Subsequently, the relative alterations in the fragmentation pattern of each subtype in the treated groups were calculated relative to the control group.

#### Embryo culture and developmental trajectory:

Naïve mouse zygote-stage embryos were injected with sperm RNA from either control, DDT-exposed or obese (OID) males in the male pro-nucleus. Briefly, injected one-cell zygotes were cultured in Advanced KSOM (Millipore, catalog #MR-101-D) at 37°C in 5% CO2 and embryonic development microscopically checked every 24 hours until harvest at 96h-post injection. Embryos were fixed or frozen and stored in −80°C. The rates of first-cleavage (zygote to two-cell embryo), two cell-stage to morula and morula to blastocyst were examined to evaluated pre-implantation embryonic development.

#### Immnunofluorescence:

Blastocyst stage embryos were transferred onto coverslips coated with concanavalin A (0.1 mg/mL) and fixed in ice-cold methanol at −20 °C for 20 min. Following fixation, embryos were washed three times in PBS and permeabilized for 30 min in 0.25% Triton X-100 in PBS. Nonspecific antibody binding was blocked by incubation in 10% fetal bovine serum (FBS) diluted in 0.1% Triton X-100 in PBS for 2 h at room temperature. Embryos were then incubated overnight at 4 °C with primary antibodies against CDX2 (1:50; Biogenex, catalog #MU392A-5UC) and OCT4 (1:200; Abcam, catalog #ab181557). After washing, primary antibodies were detected using species-appropriate Alexa Fluor 488–conjugated (1:500; Invitrogen, catalog #A32731) and Alexa Fluor 594–conjugated secondary antibodies (1:500; Thermo Fisher Scientific, catalog # A-11032). Nuclei were counterstained with DAPI using Antifade Mountant with DAPI (Invitrogen, catalog # S36964). Imaging was performed using a Leica SP8 laser-scanning confocal microscope under identical acquisition settings across experimental groups.

Placentas collected at embryonic day 13.5 (E13.5) were subjected to heat-mediated antigen retrieval in 1X sodium citrate buffer for 20 min, followed by blocking in 3% BSA for 30 min and overnight incubation at 4 °C with primary antibodies against MCT1 (1:200; Sigma-Aldrich, catalog #AB1286-I) and MCT4 (1:200; Sigma-Aldrich, catalog #AB3314P) in a humidified chamber. After PBS washes, sections were incubated for 45 min with Alexa Fluor secondary antibodies (488-conjugated, 1:600; Invitrogen, catalog #A32731 and 647-conjugated, 1:600; Abcam, catalog #ab150175). Nuclei were counterstained with DAPI (Invitrogen, catalog #S36964) and images acquired using a Leica SP8 confocal microscope. For placenta vascular area quantification, three fields per placenta were analyzed in ImageJ (Fiji), and mean values were used for statistical analysis.

#### Quantitative real time PCR validation (qRT-PCR): Quantitative real time PCR validation (qRT-PCR):

Total RNA was extracted from whole E3.5 embryos or whole E13.5 placentas using the miRNeasy Micro Kit (Qiagen, catalog #217084) according to the manufacturer’s instructions. cDNA was synthesized from total RNA using the High-Capacity cDNA Reverse Transcription Kit (Applied Biosystems, catalog #4374966). Quantitative PCR reactions were performed using PowerUp SYBR Green Master Mix (Applied Biosystems, catalog #A2572) and sequence-specific primers obtained from PrimeTime qPCR Primer Assays (Integrated DNA Technologies, IDT). Fold change was calculated from Ct values and the expression levels of specific genes were determined by normalizing these values by *GAPDH* as the endogenous control.

#### Spatial transcriptomics (ST) profiling:

ST experiments were performed using the Visium HD Platform (10x Genomics) according to the manufacturer’s protocols. Briefly, FFPE placenta sections (E13.5) were H&E-stained and imaged. Each slide was then incubated with mouse-specific probe sets for mRNA labeling, transferred using CytAssist onto a Visium slide and library generated per manufacturer’s protocol. Libraries will undergo quality control (Agilent) and sequenced on an Illumina Sequencer. For each tissue section and corresponding library, the whole-slide bright-field image and the CytAssist image were aligned manually using the Loupe Browser with landmark registration. Rectangular region of interest (ROI) containing the tissue was drawn and OMETIFF images of each ROI generated. **Spatial transcriptomics data pre-processing:** Raw sequencing data from each chip were processed independently using SpaceRanger (10x Genomics, v3.1.3). SpaceRanger was used to demultiplex reads, align them to the GRCm38 reference genome, and generate per-spot gene expression matrices along with high-resolution spatial coordinates. Quality metrics were inspected for all samples to ensure consistency across chips. To increase local spatial resolution and capture finer transcriptional gradients across tissues SpaceRanger 8-μm spatial binning resolution outputs were imported into Seurat R package (v5.3.0). Bins were filtered based on UMI counts >=100, detected gene number >= 10, and mitochondrial percentage <= 5%. All subsequent analyses were performed on the filtered datasets. Due to substantial biological and spatial heterogeneity in placental tissue architecture, and because each Visium chip contains a tissue section with distinct slicing orientation and spatial positioning, each of the four chips was processed and normalized independently using default method within Seurat. Spatial bins were clustered using the shared nearest neighbor (SNN) graph and annotated according to expression of curated canonical marker genes. Cell annotation markers are listed in Supplementary Table 5 and have been described elsewhere^56^. Differentially expressed genes among clusters were examined to support annotations. Marker expression was visualized using spatial feature plots and UMAP overlays to confirm spatial coherence and biological plausibility of assigned cell types. A total of 17 cell clusters were identified across the dataset. The number of cells per cluster per treatment condition was calculated using the annotated 8 um filtered results. **Pseudobulk differential gene expression analysis across phenotypes:** Differential gene expression was assessed using a pseudobulk strategy to preserve biological replication. Spatial scRNA-seq data from the four samples were merged in Seurat (v 5.3.0), and cells lacking phenotype annotations were removed. For each annotated cell type, raw gene counts from individual cells were summed within each sample × condition combination to generate pseudobulk profiles, yielding one count vector per biological replicate for each condition. These cluster-specific pseudobulk matrices were analyzed independently using DESeq2 (v1.40.2) with a design formula including only the treatment condition (CO-RNA, OIDRNA, DDT-RNA). DESeq2’s independent filtering was used to remove low-abundance genes, and log2 fold changes were estimated with shrinkage to improve stability. Results from all cell types were combined to produce a gene-by-cell-type matrix of treatment-associated log2 fold changes for downstream visualization and interpretation. DEG with adjusted p-value < 0.05 were considered significant. **Pathway and functional enrichment analysis:** GSEA style pathway analysis was performed using consensus ranked gene lists from each chip analysis. GO term annotations and KEGG pathway gene sets databases were used. Enriched pathways with adjusted p-value < 0.25 were considered significant. Both upregulated (activated) and downregulated (suppressed) pathways were identified for each cell type per comparison. **Imprinted gene scoring using UCell:** Curated sets of paternal and maternal imprinted genes were compiled from established imprinting databases. UCell scoring was applied to each bin within each chip to calculate enrichment scores using UCell R package (v.2.4.0). To obtain a phenotype-level summary, scores from the four chips were combined using a pseudobulk approach by averaging values across samples for each cell type and condition (CO-RNA, DDT-RNA, OID-RNA). A single heatmap was generated from these aggregated scores, and for visualization purposes values were rescaled within each cell type to highlight relative shifts in parental imprinting activity across phenotypes. **Placenta cell density proportions:** The cell density proportions of different cell types across the placenta were calculated as the number of cells of each type in a cell bin (bin8) divided by the area (in centimeters) of a specific placenta region (decidua, junctional zone, labyrinth).

#### Statistical analysis:

Statistical analyses were performed using GraphPad Prism (GraphPad Software, San Diego, CA, USA). Statistical significance was tested by student’s *t*-test or Mann-Whitney test, one or two-way ANOVA followed by Holm-Sidak’s test, Chi-square or Fisher exact test as appropriate and specified in each figure legend. Differences were considered statistically significant at P< 0.05. Unless indicated, *n* corresponds to the number of animals used in each experiment.

## Extended Data

## Data Availability

All raw sequencing data (small RNAs and spatial transcriptome) has been deposited in public databases with public accession pending (**GSE324710**, reviewer **ejmxwwoqhfmzpil**)
